# Maternal postnatal depression and child growth: a European cohort study

**DOI:** 10.1186/1471-2431-10-14

**Published:** 2010-03-12

**Authors:** Veit Grote, Torstein Vik, Rüdiger von Kries, Veronica Luque, Jerzy Socha, Elvira Verduci, Clotilde Carlier, Berthold Koletzko

**Affiliations:** 1 Institute of Social Paediatrics and Adolescent Medicine, Ludwig-Maximilians-University of Munich, Munich, Germany; 2 Department of Public Health and General Practice, Faculty of Medicine, Norwegian University of Science and Technology, Trondheim, Norway; 3 Dr von Hauner Children's Hospital, Ludwig-Maximilians-University of Munich, Munich, Germany; 4 Unitat de Recerca en Pediatria, Nutrició i Desenvolupament Humà, Universitat Rovira i Virgili, Tarragona, Spain; 5 Children's Memorial Health Institute, Warsaw, Poland; 6 Department of Pediatrics, San Paolo Hospital, University of Milan, Milan, Italy; 7 Université Libre de Bruxelles Childrens' Hospital, Bruxelles, Belgium

## Abstract

**Background:**

Previous studies have reported postpartum depression to be associated with both positive and negative effects on early infant growth. This study examined the hypothesis that maternal postnatal depression may be a risk factor for later child growth faltering or overweight.

**Methods:**

A total of 929 women and their children participating in a European multicenter study were included at a median age of 14 days. Mothers completed the Edinburgh postnatal depression scale (EPDS) at 2, 3 and 6 months after delivery. EPDS scores of 13 and above at any time were defined as maternal depression. Weight, length, triceps and subscapular skinfold thicknesses were measured, and body mass index (BMI) were calculated when the children were two years old and converted to standard deviation scores based on the WHO Multicentre Growth Reference Study (MGRS).

**Results:**

Z-scores for weight-for-length at inclusion of infants of mothers with high EPDS scores (-0.55, SD 0.74) were lower than of those with normal scores (-0.36, SD 0.74; p = 0.013). BMI at age 24 months did not differ in the high (16.3 kg/m2, SD 1.3) and in the normal EPDS groups (16.2 kg/m2, SD 1.3; p = 0.48). All other anthropometric indices also did not differ between groups, with no change by multivariate adjustment.

**Conclusions:**

We conclude that a high maternal postnatal depression score does not have any major effects on offspring growth in high income countries.

## Background

Postpartum depression has a prevalence of about 10-15% [1] with a high variation between populations [2]. Risk factors include stressful life events, unemployment, marital conflict, lack of social support, low income, low education, previous history of depression and caesarean section [3,4].

Numerous parenting difficulties are reported to exist in affected parents [5,6] with associated long term emotional, cognitive, and behavioural problems in the offspring [7]. Mothers who were depressed were more likely to have a less healthy lifestyle and to engage in less healthy feeding and sleep practices with their infant [8,9]. Depressed mothers might have inadequate nutritional intakes [10], and they tend to stop breastfeeding earlier than non-depressed mothers [11-13].

It is also well recognised that postpartum depression can have negative effects on early infant growth [14-18], a problem that might be more pronounced in low-income countries with less favourable environments [18]. Surkan et al [19], on the other hand, recently observed a more rapid weight gain in children of mothers affected by postpartum depression in a Brazilian low-income cohort. They speculated that the same mechanism, disturbances in the feeding behaviour of depressed mothers, could affect both tails of the growth distribution, leading either to stunting or obesity.

In a European birth cohort we explored if weight, length or skinfold thicknesses differed at two years of age between children of mothers with high postnatal depression symptom scores and children of mothers with normal scores, and if postnatal depression had different effects on the lower and upper tails of the distribution of anthropometric measures.

## Methods

### Design and study population

This study uses data from a randomized controlled multicenter study assessing the effect of higher or lower protein formula on overweight later in childhood. Details of the study have been published [20]. In short, participants were recruited at 11 study sites in five countries (Belgium; Germany; Italy; Poland; Spain). Eligible for study participation were healthy, singleton, term infants born between 1^st ^October 2002 and 31^st ^July 2004. Infants were enrolled during the first eight weeks of life and were either randomized to a high or low protein formula group, or were included in an observational group of breastfed infants. In this study, data from children in the interventional group and the observational group were combined.

At visits 2, 3 and 6 months after delivery, the mothers were asked to complete the Edinburgh postnatal depression scale (EPDS) [21]. Of originally 1678 recruited children, 930 children were still in the study at 2 years of age and had also anthropometric measurements taken at that time point. Of these children an EPDS score were obtained in 901 children at two months, in 905 at three, in 879 at six and in 929 children any time point after birth.

### Methods

The EPDS is a 10 item self-report instrument [21]. Each item is scored from 0 to 3, and the total score equals the sum of the ten items. Validated scores of 13 or more are used to identify mothers at risk of being depressed [21,22]. However, for research purposes a cut-off score of 9 has also been proposed [15].

Data on the course of pregnancy, birth weight, medical history, lifestyle, behaviour and socio-economic background were recorded at recruitment.

Measurements of infant growth were obtained at study entry, and when the infants were 3, 6, 12 and 24 months old, and included weight, length/height, triceps and subscapular skinfold thickness. Based upon these measurements, BMI values (body weight (kg) divided by the squared value of height (m)) were calculated. Both absolute values and z-scores based upon WHO growth standards [23] were used in the analyses. Birth weight and length were obtained from hospital data.

All study sites used the same equipment (Seca 727 scales, Hamburg, Germany; Ellard PED LB 35-107 X, Ellard Instruments, Monroe, USA). Written standard operating procedures for measures and calibration were established, and repeated training sessions for all study personnel and site visits by the team of the principal investigator and external experts were performed.

### Ethics

The study was approved by the ethics committees of all study centers. Written informed parental consent was obtained for each infant.

### Statistical analyses

Anthropometric measurements were expressed as z-scores relative to the growth standards of the World Health Organization for breastfed children [23]. Z-scores were calculated using WHO programs http://www.who.int/childgrowth/software/en/. The primary endpoints were length and weight at 24 months, which were expressed as standard deviation scores (z-score) of length-for-age and weight-for-length. Weight-for-length shows less variation than weight-for-age and is a better descriptor of body composition in children than weight. Postnatal, maternal depression as defined by EPDS scores of 13 or more at any time were the main determinant, but we also explored the data using an EPDS cut-off score of 9 and looked for a potential dose-response relationship by examining linear trends over 4 categories of EPDS scores (<9, 10-12, 13-15, >15).

The chi-square test was used to analyse differences in proportions between groups. Comparisons of mean values were done using independent samples t-test.

For multivariable analysis we used linear regression and quantile regression as suggested by Beyerlein et al. [24] to test if differences in z-scores in the higher or lower depression group differed between the 5th, 15th, 50th, 85th and 95th percentile.

All multivariable analyses were adjusted for the respective anthropometric baseline measurement at inclusion into the study. Furthermore, feeding type and country were included in all models. To appreciate the potential confounding of other variables, we looked at sensible changes after variable inclusion on the effect size of maternal depression and its confidence interval. As potential confounders we considered gender, pregnancy not wished, stress in pregnancy, caesarean section, mothers education, mothers BMI, smoking status, birth order of the child, mothers age, mothers partnership status (married, single) and mothers age.

We also applied multilevel linear growth models and piecewise-linear-random-coefficient models as described by Singer and Willet [25] and Fitzmaurice et al. [26] to model growth differences between the lower and higher protein formula group using all available measurements from baseline to 24 months. Both models account for the correlated data structure due to the repeated measurements and use the exact age of measurement. The piecewise-linear-random-coefficient model was chosen to analyse the age-dependent effect of maternal depression on the anthropometric outcome. The idea of the model is to split the time in fixed segments with different slopes in each segment, in contrast to the usual multilevel linear growth model which uses one slope over the whole analysis time. The choice of the time segments (0-3 months, 3-6 months, 6-12 months and 12-24 months) for this model is based on the measurement points as planned per protocol. Statistical significance of differences between trajectories of the study groups in the piecewise-linear-random-coefficient model was assessed by 95%-prediction-bands. If the 95%-prediction-bands of one group (e.g. lower protein) does not overlap with the average trajectory of the other study group (i.e. maternal depression - EPDS > 13), there is a significant difference between these trajectories with a 5%-probability error.

All data were analyzed using Stata 9.2.

## Results

Background data of the 929 children comprised in the analysis are listed in Table 1. One-hundred two (11.0%) mothers had an EPDS score of 13 or more at any time, indicating maternal depression; at 2 month 6.9% of the mothers, at 3 month 4.3% and at 6 months 4.0% mothers had high EPDS scores. Eighteen (1.7%) mothers had prolonged symptoms of depression, i.e. high EPDS scores at 2 and/or 3 months and at 6 months. The prevalence of high EPDS scores varied significantly between the 5 participating countries and ranged from 6-8% in Germany and Spain to 13-16% in Belgium, Poland and Italy.

**Table 1:** Background data and WHO z-score for weight-for length (WFL) of 929 children and their mother with available Edinburgh postnatal depression scale (EPDS) and anthropometry at 24 months.

		n	%/mean(SD)	z-score weight-for length at 24 months	p-value
Total		929	100.0	0.26 (0.91)	
Ever EPDS >= 13 at 2, 3 or 6 months	<13	827	89.0	0.25 (0.91)	0.6431
	>=13	102	11.0	0.30 (0.91)	
EPDS >= 13 at 2 months	<13	839	93.1	0.25 (0.90)	0.5053
	>=13	62	6.9	0.33 (1.02)	
EPDS >= 13 at 2 or 3 months and 6 months	<13	903	98.3	0.25 (0.91)	0.2963
	>=13	16	1.7	0.01 (0.83)	
					
Country	Germany	143	15.4	0.18 (0.89)	<0.001
	Belgium	116	12.5	0.38 (0.86)	
	Italy	265	28.5	-0.04 (0.88)	
	Poland	159	17.1	0.36 (0.90)	
	Spain	246	26.5	0.21 (0.99)	
					
Gender	male	449	48.3	0.29 (0.85)	0.2552
	female	480	51.7	0.22 (0.97)	
					
Birth order	1st child	537	57.9	0.28 (0.92)	0.5814
	2nd child	301	32.5	0.23 (0.92)	
	>2nd child	89	9.6	0.18 (0.82)	
					
Birth weight	<3130 g	313	33.7	0.29 (0.85)	0.0017
	3130 - 3450 g	314	33.8	0.11 (0.88)	
	>3450 g	302	32.5	0.37 (0.98)	
					
Study formula	lower protein	313	33.7	0.37 (0.93)	0.0197
	higher protein	319	34.3	0.18 (0.86)	
	breastfed	297	32.0	0.21 (0.93)	
					
Single mother	yes	38	4.1	0.26 (0.91)	0.3928
	no	889	95.9	0.13 (0.90)	
					
Pregnancy not wished	yes	73	7.9	0.24 (0.91)	0.2041
	no	852	92.1	0.38 (0.92)	
					
Mother frequently experienced stress during pregnancy	yes	139	15.0	0.25 (0.91)	0.8096
	no	789	85.0	0.27 (0.94)	
					
Caesarean section	yes	197	21.3	0.22 (0.92)	0.0487
	no	728	78.7	0.37 (0.89)	
					
Mother's education level (ISCED)	no/low	198	21.4	0.32 (0.94)	0.3906
	middle	472	50.9	0.22 (0.91)	
	high	257	27.7	0.25 (0.87)	
					
Smoking of mother 3 months prior to or during pregnancy	yes	324	35.0	0.21 (0.93)	0.0362
	no	602	65.0	0.34 (0.87)	
					
Smoking of mother beyond 12th week of pregnancy	yes	173	18.7	0.24 (0.91)	0.1780
	no	754	81.3	0.34 (0.90)	
					
BMI mother	<20	163	18.3	0.25 (0.86)	0.1585
	20-<25	482	54.0	0.25 (1.00)	
	25-<30	183	20.5	0.19 (0.93)	
	>=30	64	7.2	0.49 (0.99)	
					
Mother's age	<28	253	27.3	0.27 (0.90)	0.9231
	28-<33	363	39.1	0.26 (0.89)	
	33-44	312	33.6	0.24 (0.96)	

Six-hundred thirty-two (68%) children were included in the formula-fed intervention arm of the study, and 297 (32%) children were fully breastfed until 4 months of age. More than 7% of the mothers were obese and almost 19% of the mothers smoked during pregnancy beyond the first trimester (Table 1).

Most anthropometric measures of children differed significantly between participating countries, for instance BMI ranged from 15.8 kg/m2 in Germany to 16.4 kg/m2 in Italy. Also gender, birth order, smoking of the mother, caesarean section, unwished pregnancy, and type of feeding had some significant association with anthropometric measures.

There was some indication that children in the high EPDS group were lighter at birth (3237 g, SD 352 versus 3301 g, SD 346; p = 0.081) and at inclusion (z-score weight -0.55, SD 0.74 versus -0.36, SD 0.74; p = 0.013). However, Table 2 shows that weight, length, triceps or subscapular skin-fold thickness as well as weight-for-length and BMI did not differ between children of mothers with high and normal EPDS scores at 24 months of age.

**Table 2:** Outcome at age 24 months in children of mothers with normal (<13) or high (≥13) scores on the Edinburgh postnatal depression scale (EPDS).

EPDS	<13	≥13		
	N = 827	N = 102	Estimated difference in z-score	
	mean (SD)	mean (SD)	Baseline adjusted	Fully adjusted*
Age at exam (Months)	24.2 (0.6)	24.2 (0.5)		
Weight (kg)	12.4 (1.4)	12.4 (1.4)		
z-score	0.34 (0.91)	0.35 (0.93)	0.08 (-0.10, 0.26)	0.01 (-0.18, 0.19)
Length (cm)	88.0 (3.3)	87.8 (3.1)		
z-score	0.22 (1.01)	0.17 (0.99)	0.03 (-0.16, 0.23)	-0.10 (-0.29, 0.10)
Triceps skinfold thickness (mm)	8.9 (2.1)	9.1 (2.2)		
z-score	0.52 (1.11)	0.62 (1.14)	0.05 (-0.23, 0.33)	0.09 (-0.21, 0.39)
Subscap skinfold thickness (mm)	6.6 (1.6)	6.9 (1.8)		
z-score	0.29 (1.13)	0.48 (1.11)	0.13 (-0.14, 0.41)	0.14 (-0.16, 0.44)
Weight-for length				
z-score	0.25 (0.91)	0.30 (0.91)	0.08 (-0.10, 0.26)	0.01 (-0.18, 0.19)

* adjusted by country, feeding type, single mother, mother's age, birthorder, bmi of mother, stress in pregnancy, caesarean section, pregnancy not wished, and respective anthropometric measurement at baseline, smoking

Multivariable analyses indicated that none of the potential confounders, including country, had a significant effect on the effect estimate of maternal depression. Thus, the effect size was basically unchanged when considering all potential confounders (Table 2). The results were also basically unchanged when we restricted the analyses to the three countries with a prevalence of high EPDS scores (Poland, Italy and Belgium), lowering the EPDS cut-off of maternal depression to 9, restricting the children to those with depressed mothers at 2 or 3 months only, or looking at earlier time points (6 or 12 months) of anthropometric measurements. Looking at the effects of EPDS categorised in 4 severity levels, we did not find a dose-response relationship on anthropometric measures.

Only for weight - not for length, weight-for-length or BMI - there was a significant difference in the multilevel linear growth model between the children of mothers with an elevated EPDS score and those of mothers with normal scores, with the latter having a higher weight. However, the model also indicated that this effect was not constant over the whole 24 months period. The effect of maternal depression on weight was already present at inclusion into the study and was more or less constant until the 12 months measurement and decreased thereafter (additional file 1).

**Figure 1:**
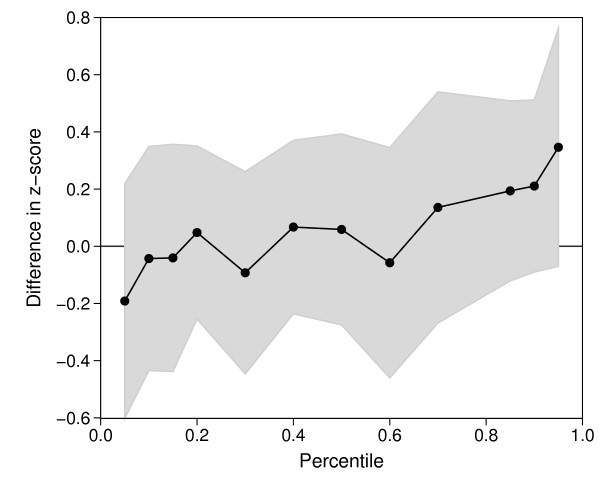
**Point estimates and 95% confidence bounds for differences in WFL-z-score at 24 months of age between children of mothers with high (>=13) and low (<13) EPDS score adjusted by potential confounders**. The connected dots represent the estimated difference at specific WFL percentiles (0.05, 0.1, 0.15, 0.2 to 0.8, 0.85, 0.9 and 0.95 percentile) obtained from the multivariable quantile regression model.

Since we did not find any effect of maternal depression on the mean of any anthropometric measure, we also looked at possible effects on the lower and upper tails of their distributions (Table 3). As exemplified for weight-for-length in Figure 1 we could not discern any differences between the high and low EPDS group over the whole BMI or any other anthropometric measurement distribution.

**Table 3:** Difference between children of mothers with higher and lower (<13) EPDS score at different percentiles of respective anthropometric measure at 24 months.

	Difference between children of mothers with higher and lower (<13) EPDS score at different percentiles of respective anthropometric measure at 24 months			
Anthropometric measure	5th perc.	15th perc.	85th perc.	95th perc.
Weight-for-age z-score (WHO)	-0.10 (-0.62, 0.41)	0.09 (-0.19, 0.37)	0.01 (-0.28, 0.31)	-0.00 (-0.36, 0.36)
Length/height-for-age z-score (WHO)	0.01 (-0.45, 0.47)	-0.03 (-0.48, 0.41)	-0.14 (-0.50, 0.21)	-0.02 (-0.53, 0.49)
BMI-for-age z-score (WHO)	-0.02 (-0.39, 0.35)	-0.04 (-0.37, 0.30)	0.20 (-0.19, 0.58)	0.22 (-0.35, 0.78)
Weight-for-length z-score (WHO)	-0.19 (-0.60, 0.21)	-0.04 (-0.34, 0.26)	0.19 (-0.16, 0.55)	0.35 (-0.14, 0.83)
Subscapular skinfold-for-age z-score (WHO)	0.19 (-0.27, 0.65)	-0.02 (-0.50, 0.47)	-0.01 (-0.57, 0.56)	-0.16 (-0.98, 0.65)
Triceps skinfold-for-age z-score (WHO)	0.18 (-0.31, 0.67)	0.01 (-0.40, 0.42)	0.22 (-0.29, 0.73)	0.04 (-0.28, 0.36)

## Discussion

In this cohort study we found no effect of postnatal maternal depression on the child's BMI or other anthropometric indices in the first two years of life. Thus, the results do not support the hypothesis that high postnatal depression scores may be a risk factor for under- or overweight in childhood.

Several studies have found negative effects on early infant growth in low-income countries [11,17,18,27-29]. However, results from high-income countries like the US or the UK are inconsistent [14-16], with the largest study [30] including over 12, 000 children showing no association between maternal depression and failure to thrive over the first nine months of life. Wright et al suggested that the potential effect of depressive symptoms might be only transitional, seen at 4 month of life but no longer at 12 months of age [14]. Therefore, we also analysed anthropometric data at 6, 12 and 24 months of age but did not find any meaningful effects at earlier time points either. Nevertheless, we see some effect of maternal depression on child weight, as the children of depressed mothers are lighter at birth. Thus, one could speculate that mothers with postnatal depression are somewhat different from other mothers, and these differences might influence foetal growth.

It has been suggested that there might be a dose-response relationship, given that children of mothers with prolonged depressive symptoms had lower weight gain in one study [16]. In our population, only 16 (1.7%) of mothers had documented prolonged depressive symptoms, and hence we were unable to adequately analyse a dose-response relationship.

Strengths of the present study were the prospective design, the inclusion of 5 different European countries, the standardized and comprehensive assessments of growth, and that neither participants nor examiners were aware of the hypothesis tested here. A cut-off level of 13 on the EPDS has been proposed as the appropriate clinical cut-off value [21,22], whereas other investigators have proposed a cut-off at nine for research purposes [15]. We analysed our data using both cut-off values, but the results were essentially the same.

The proportion of women with high EPDS scores varied between countries. Whereas the prevalence rates in Germany and Spain were lower, the rates in Poland, Italy and Belgium were comparable to prevalence rates reported from a number of other countries [22,31]. When we restricted the analyses to the latter three countries, the results of our analysis were basically unchanged. The effects of maternal depression did not differ between the countries. However, the observed heterogeneity in prevalence of maternal depressive symptoms points to the fact that the EPDS may not be an equally valid screening tool across all settings and contexts [32] and that postnatal maternal depression is strongly influenced by cultural background [2].

The present study had sufficient statistical power to detect a difference in weight of approximately 400 gram (or a difference in SD of 0.3), corresponding to a difference of 3% in weight. Thus, we cannot exclude that we may have missed smaller effects of postnatal depression on growth in early childhood. Given that an odds ratio of 1.87 (95% CI 1.10 - 3.18) for obesity in 14 to 16 year old Brazilian adolescents was found for every 1 SD change in weight-for-length gain in the meta-analysis on effects of early weight gain on later obesity by Monteiro et al [33], smaller differences than 0.3 SD may not be very relevant from a public health perspective.

## Conclusions

Several events in early life, such as duration of breastfeeding, prenatal smoking exposure and poor growth have been found to be associated with increased risk of overweight and obesity in the offspring. Our results suggest that postnatal depression may not carry such risk, at least not in a European population.

## Competing interests

The authors declare that they have no competing interests.

## Authors' contributions

All authors read and approved the final manuscript. VG: data management and analysis, writing of manuscript. TV: analysis, writing of manuscript. RvK: study concept, critical reading of manuscript. VL: recruitment, conduct of study, data entry, critical reading of manuscript. JS: coordination of study, critical reading of manuscript. EV: recruitment, conduct of study, data entry, critical reading of manuscript. CC: recruitment, conduct of study, data entry, critical reading of manuscript. BK: initiator and principal investigator of study, study concept, coordination of study, writing of manuscript. European Childhood Obesity Trial Study Group: Members of the group were responsible for planning, recruitment, conduct of study, and data entry.

## Pre-publication history

The pre-publication history for this paper can be accessed here:

http://www.biomedcentral.com/1471-2431/10/14/prepub

## Supplementary Material

Additional file 1 **The effect of maternal depression on weight**. Mean trajectories of weight by children of depressed (EPDS >= 13) and non-depressed mothers over the first 24 months of life obtained by piecewise-linear-random-coefficient model. Click here for file
