# Supplementary figures and images for: Maternal postnatal depression and child growth: a European cohort study
Source: BMC Pediatr. 2010 Mar 12;10:14. doi: 10.1186/1471-2431-10-14 (PMC2850333; doi:10.1186/1471-2431-10-14)

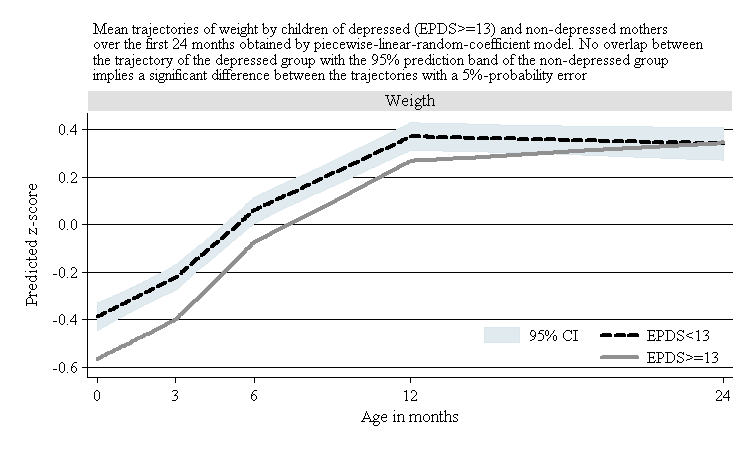

Supplement: Additional file 1 — The effect of maternal depression on weight. Mean trajectories of weight by children of depressed (EPDS >= 13) and non-depressed mothers over the first 24 months of life obtained by piecewise-linear-random-coefficient model. [file 1471-2431-10-14-S1.PNG]
